# Roflumilast improves corticosteroid resistance COPD bronchial epithelial cells stimulated with toll like receptor 3 agonist

**DOI:** 10.1186/s12931-015-0179-5

**Published:** 2015-02-05

**Authors:** Javier Milara, Anselm Morell, Bea Ballester, Celia Sanz, Jose Freire, Xiaozhong Qian, Maggie Alonso-Garcia, Esteban Morcillo, Julio Cortijo

**Affiliations:** Clinical Research Unit, University General Hospital Consortium, Valencia, Spain; CIBERES, Health Institute Carlos III, Valencia, Spain; Pharmacy Department, Fundación de Investigación, University General Hospital Consortium, Avenida tres cruces s/n, Valencia, E-46014 Spain; Department of Pharmacology, Faculty of Medicine, University of Valencia, Valencia, Spain; Research Foundation, University General Hospital Consortium, Valencia, Spain; Faculty of Biomedic Sciences, European University of Madrid; affiliated center of Valencia, Valencia, Spain; Forest Research Institute, Jersey City, NJ USA

**Keywords:** Roflimilast, Corticosteroid resistance, Toll like receptors, COPD, Viral exacerbation

## Abstract

**Background:**

Chronic obstructive pulmonary disease (COPD) is characterised by chronic pulmonary inflammation punctuated by periods of viral exacerbations. Recent evidence suggests that the combination of roflumilast with corticosteroids may improve the compromised anti-inflammatory properties of corticosteroids in COPD. We analyzed differential and combination anti-inflammatory effects of dexamethasone and roflumilast N-oxide in human bronchial epithelial cells (HBECs) stimulated with viral toll like receptor (TLR) agonists.

**Methods:**

Lung tissue and HBECs were isolated from healthy (n = 15), smokers (n = 12) and smokers with COPD (15). TLR3 expression was measured in lung tissue and in HBECs. IL-8 secretion was measured in cell cultures after TLR3 stimulation with poly I:C 10 μg/mL.

**Results:**

We found that TLR3 expression was increased by 1.95 fold (protein) and 2.5 fold (mRNA) in lung tissues from smokers with COPD and inversely correlated with lung function. The TLR3 agonist poly I:C 10 μg/mL increased the IL-8 release in HBECs that was poorly inhibited by dexamethasone in smokers (24.5%) and smokers with COPD (21.6%). In contrast, roflumilast showed similar inhibitory effects on IL-8 release in healthy (58.8%), smokers (56.6%) and smokers with COPD (50.5%). The combination of roflumilast N-oxide and dexamethasone showed additive inhibitory effects. Mechanistically, roflumilast N-oxide when combined with dexamethasone increased the expression of MKP1, and enhanced the inhibitory effects on phospho-p38, AP1 and NFκB activities which may explain the additive anti-inflammatory effects.

**Conclusions:**

Altogether, our data provide *in vitro* evidence for a possible clinical utility to add roflumilast on top of inhaled corticosteroid in COPD.

## Background

Chronic obstructive pulmonary disease (COPD) is characterized by irreversible airflow obstruction, inflammation, and a progressive decline in lung function [1]. The primary cause of COPD is chronic exposure to cigarette smoke, which leads to airway inflammation and remodeling, thus increasing airflow limitation.

The current first-line maintenance treatment for COPD involves the use of bronchodilators, including long-acting muscarinic antagonists (LAMAs) and long-acting beta agonists (LABAs), in combination with inhaled corticosteroids in those patients with severe COPD who are at risk of exacerbations. However, in contrast to other inflammatory diseases, corticosteroids are less effective in improving lung function and have little or no effect on controlling the underlying chronic inflammation in COPD patients [2]. The poor anti-inflammatory properties of corticosteroids in COPD have increased the development of other anti-inflammatory drugs. This is the case of roflumilast, the first phosphodiesterase–4 (PDE4) inhibitor approved for COPD. It is indicated as a treatment to reduce the risk of COPD exacerbations associated with chronic bronchitis in patients with severe COPD and a history of exacerbations. In preclinical models, roflumilast and its active metabolite, roflumilast N-oxide, have been shown to inhibit a broad spectrum of inflammatory cytokines and reactive oxygen species (ROS) in different inflammatory and non-inflammatory cells relevant to COPD [3].

Exacerbations of COPD are the major cause of morbidity and mortality and are associated with accelerated decline in lung function and progression of the disease [4].

Respiratory RNA virus infections such as human rhinovirus (HRV), respiratory syncytial virus (RSV) and influenza virus are common causes of exacerbations of COPD [4]. In this regard, HRV-induced lung inflammation has been recently shown to be corticosteroid resistant [5,6]. Furthermore, neutrophilic inflammation in chronic cigarette smoking mice exacerbated with influenza virus infection was resistant to dexamethasone [7]. Similarly, the toll like receptor 3 (TLR3) agonist, poly I:C, induced a corticosteroid resistant neutrophil airway inflammation in mice [8]. Current data suggests that roflumilast has anti-inflammatory effects on RSV-infected bronchial epithelial cells [9] and reverses corticosteroid resistance in neutrophils from COPD patients *in vitro* [10].

Viral exacerbations in patients with COPD are considered to be caused by inflammatory responses that overwhelm the protective anti-inflammatory defenses [4]. In fact, viral exacerbations increased neutrophil counts in the bronchial walls and in bronchoalveolar lavage fluid from COPD patients mainly through the release of neutrophil chemoattractant inflammatory cytokines IL-8 or LTB4 by infected airway epithelial cells [11-14].

The double-stranded (ds) and single-stranded (ss) RNA generated during RNA virus infection activates TLR3 and TLR7/8, respectively, which enhance inflammatory and anti-viral responses as part of the host innate immune defense [15]. The expression of TLR3 has been detected in immune cells such as macrophages, natural killer cells, CD8^+^ T cells, and dendritic cells, and non-immune cells such as airway smooth muscle cells, airway epithelial cells, and endothelial cells [16-19]. The expression of TLR7/8 was first detected on dendritic cells and later in leukocytes, lymphocytes, endothelial and airway epithelial cells [20]. Despite the key role of viral exacerbations on COPD progression, the expression levels and distribution pattern of TLR3 and TLR7/8 in inflammatory cells and lung tissue of COPD patients are not sufficiently characterized and the information currently available is contradictory [17,19].

The purpose of the current study was to characterize the expression and distribution of TLR3, TLR7, and TLR8 in lung tissue from non-smokers, smokers and smokers with COPD and to analyze the differential effects of roflumilast N-oxide *versus* corticosteroids and their potential additive or synergistic anti-inflammatory effect in bronchial epithelial cells stimulated with TLR3/7/8 agonists. Results from this study may be of potential value to understanding the clinical benefits of combining roflumilast and corticosteroids for COPD treatment [21].

## Methods

### Patients

A total of 15 non-smoking controls, 12 current smokers without COPD, and 15 current smokers with COPD were included in the study. COPD patients were diagnosed according to the GOLD guidelines [22]. All lung tissues studied were taken from lung explants of non-smoking subjects in the transplant program and from uninvolved lung tissue from smokers without COPD or with COPD during lobectomy/wedge resection for malignant lesions in the Thoracic Surgery and Respiratory Unit, University General Hospital Consortium, Valencia, Spain, between 2010 and 2014. Samples of distal lung, located as far as possible from the tumor, were chosen for the study. All pulmonary function tests were performed within 3 months before surgery or taken from the clinical history. Clinical data from all patients (see Table 1) were examined for possible co-morbidity. Inclusion criteria comprised either non-smokers or current smokers with or without COPD who were free of symptoms of upper respiratory tract infection and were not receiving antibiotics perioperatively. After selection based on lung function, all lung tissue samples chosen for the study were checked histologically using the following exclusion criteria: (1) presence of tumor, (2) presence of post stenotic pneumonia, and (3) fibrosis of lung parenchyma. The study protocol was approved by the local research and independent ethics committee of the University General Hospital of Valencia. Informed written consent was obtained from each participant or their legal representative.

**Table 1:** **Clinical characteristics of patients**

	**Non-smokers (n = 15)**	**Smokers (n = 12)**	**Smokers with COPD (n = 15)**
Sex (Female/Male)	7/8	3/9	3/12
Age (years)	60 [50–72]	60 [47–68]	55 [49–60]
Tobacco consumption, pack-year	0	40 [20–56]*	55 [34–83]*
FEV1, % predicted	92 [76–96]	87 [83–97]	73 [53–79]^‡^
FVC, % predicted	97 [72–107]	90 [86–99]	96 [84–114]
FEV1/FVC	78 [75–84]	80 [77–83]	61 [55–67]^‡^
PaO_2_, mmHg	94 [89–99]	85 [81–90]^†^	80 [78–83]^‡^
PaCO_2_ mmHg	41 [34–47]	44 [37–48]	47 [42–54]^†^

COPD: chronic obstructive pulmonary disease; FEV1: forced expiratory volume in one second; FVC: forced vital capacity; Pack-yr = 1 year smoking 20 cigarettes-day; PaO_2_: oxygen tension in arterial blood; PaCO_2_: carbon dioxide tension in arterial blood; Data are the median [interquartile range]. * P < 0.05 compared with nonsmokers. ^†^P < 0.05 compared with nonsmokers and smokers without COPD. ‡ P < 0.05 compared with nonsmokers and smokers without COPD.

### Isolation of primary bronchial epithelial cells and cell culture

Human bronchial epithelial cells (HBECs) from small bronchi were isolated as previously outlined [23]. Small pieces of human bronchi (0.5–1 mm internal diameter) were excised from microscopically normal lung areas, carefully dissected from lung parenchyma and plated on collagen-coated culture dishes (10 μg/cm^2^ rat type I collagen; Sigma) in bronchial epithelial growth medium (BEGM), comprising bronchial epithelial basal medium (BEBM) supplemented with bovine pituitary extract (52 μg/ml, hydrocortisone 0.5 μg/ml), human recombinant epidermal growth factor ([EGF] 25 ng/ml), epinephrine (0.5 μg/ml), transferrin (10 μg/ml), insulin (5 μg/ml), retinoic acid (50 nM), triiodo-L-thyronine (6.5 ng/ml), gentamycin (40 μg/ml), amphotericin B (50 ng/ml), and bovine serum albumin (1.5 μg/ml). Small bronchi were oriented with the epithelial layer in contact with the culture plate. After a period of ~1–2 weeks, bronchial epithelial cells were observed around the bronchi. After trypsinization (passage 1), cells were cultured accordingly for different experiments. All the experiments performed in this study with primary HBEC were done on monolayer cultures. The identity of the monolayer as bronchial epithelial cells was confirmed using morphological criteria and immunofluorescence for cytokeratin 5 (KRT5) as well as later use of *in vitro* differentiation in air-liquid interface as pseudo-stratified bronchial epithelium with basal cells, ciliated cells, columnar, and goblet cells (data not shown). Cell viability was assessed by vital trypan blue exclusion analysis using the Countness® automated cell counter (Life Technologies, Madrid, Spain). Cell viability was >98% in all cell cultures.

The bronchial epithelial BEAS2B cell line was obtained from American Type Culture Collection and cultured in BEGM media with supplements (Lonza, Madrid, Spain) on collagen-coated culture dishes (10 μg cm^-2^; rat type I collagen) at 37°C with 5% CO_2_ in humidified air. The culture medium was replaced every 48 hours.

### Immunohistochemistry

For immunohistochemical analysis of human pulmonary tissue from non-smokers, smokers, and smokers with COPD, tissues were fixed, embedded in paraffin, cut into sections (4–6 μm), and stained with haematoxylin, as reported previously [24]. The sections were incubated with rabbit anti-human TLR3 polyclonal antibody (diluted 1:250; Bioss, Woburn, USA), rabbit anti-human TLR7 polyclonal antibody (1:250; Novus Biologicals®, Madrid, Spain), rabbit anti-human TLR8 polyclonal antibody (1:250; Novus Biologicals®, Madrid, Spain) for 24 hours at 4°C. A secondary anti-rabbit antibody (1:100; Vector Laboratories, Burlingame, CA) with avidin-biotin complex/horseradish peroxidase (HRP) was used for immunohistochemistry. The non-immune IgG isotype control was used as negative control. All stained slides were scored by a pathologist under a Nikon Eclipse TE200 (Tokio, Japan) light microscope and representative photographs taken (10 slices per patient) as previously outlined [25]. Staining intensity was analyzed in alveolar macrophages and bronchial epithelium of small bronchi. Staining intensity for TLR3, TLR7, and TLR8 antibodies was scored on a scale of 0–3: 0, negative; 1, weak; 2, moderate; or 3, strong immunoreactivity. The percentage of cells positive for TLR3, TLR7 and TLR8 antibodies in alveolar macrophages and within bronchial epithelium was scored on a scale of 1–4 as follows: 1, 0–25% cells positive; 2, 26–50% positive; 3, 51–75% positive; and 4, 76–100% positive. The score of the staining intensity and the percentage of immunoreactive cells were then multiplied to obtain a composite score ranging from 0 to 12.

### Western blot

Western blot analyses were performed to detect changes in TLR3, TLR7, and TLR8 expression in lung tissue from non-smokers, smokers, and smokers with COPD. The Bio-Rad assay (Bio-Rad Laboratories Ltd., Herts, UK) was utilized to quantify the level of protein in each sample to ensure equal protein loading. Proteins were separated according to molecular weight by SDS-PAGE. Briefly, 15 μg of denatured protein and a molecular weight protein marker (Bio-Rad Kaleidoscope marker; Bio-Rad Laboratories) were loaded onto an acrylamide gel consisting of a 5% acrylamide stacking gel stacked on a 10% acrylamide resolving gel and electrophoresed at 100 V for 1 hour. Proteins were transferred to a polyvinylidene difluoride (PVDF) membrane using a wet blotting method. The membrane was blocked with 5% Marvel in PBS containing 0.1% Tween20 (PBS-T), probed with a rabbit anti-human TLR3 polyclonal antibody (1:1000; Bioss, Woburn, USA), rabbit anti-human TLR7 polyclonal antibody (1:1000; Novus Biologicals®, Madrid, Spain), rabbit anti-human TLR8 polyclonal antibody (1:1000; Novus Biologicals®, Madrid, Spain), and normalised to total mouse anti-human β-actin monoclonal antibody (1:1000; Sigma). Labeled proteins were detected using enhanced chemiluminescence methods and reagents (ECL plus; Amersham GE Healthcare, Buckinghamshire, UK). Densitometry of films was performed using the Image J 1.42q software (available at http://rsb.info.nih.gov/ij/, USA) and results are expressed as the ratio of the densitometry of the endogenous control β-actin.

### Real Time RT-PCR

Total RNA was isolated from lung parenchyma, primary HBECs, and BEAS2B bronchial epithelial cells with the TriPure® Isolation Reagent (Roche, Indianapolis, USA). The integrity of the extracted RNA was confirmed with the Bioanalyzer (Agilent, Palo Alto, CA, USA). Reverse transcription was performed in 300 ng of total RNA with TaqMan reverse transcription reagents kit (Applied Biosystems, Perkin-Elmer Corporation, CA, USA). cDNA was amplified with specific, pre-designed primer sets for MKP1, MIF, HDAC2, TLR3, TLR7, TLR8, and TRIF and the housekeeping gene GAPDH (Applied Biosystems) in a 7900HT Fast Real-Time PCR System (Applied Biosystems) using Universal Master Mix (Applied Biosystems). Relative quantification of each transcript was determined with the 2^-ΔΔCt^ method using GAPDH as endogenous control and normalized to the non-smoker or control groups.

### Preparation of cigarette smoke extract solutions

Cigarette smoke extract (CSE) was prepared as previously outlined [26]. Briefly, the smoke of a research cigarette (2R4F; Tobacco Health Research, University of Kentucky, KY, USA) was generated by a respiratory pump (Apparatus Rodent Respirator 680; Harvard, Germany) through a puffing mechanism similar to the human smoking pattern (3 puffs/min; 1 puff volume of 35 ml; each puff duration lasting 2 seconds with 0.5 cm above the filter) and was bubbled into a flask containing 25 ml of pre-warmed (37°C) Roswell Park Memorial Institute (RPMI)-1640 culture medium. The CSE solution was sterilized by filtration through a 0.22 μm cellulose acetate sterilizing system (Corning, USA). The resulting CSE solution was considered 100% CSE and was used within 30 minutes of preparation. CSE 10% approximately corresponds to the exposure associated with smoking of two packs per day [27]. The quality of the prepared CSE solution was assessed based on the absorbance at 320 nm, which is the specific light absorption wavelength of peroxynitrite. Stock solutions with an absorbance value of 3.0 ± 0.1 were used. To test for cytotoxicity and apoptosis due to CSE, BEAS2B cells were treated with CSE concentrations of up to 5% for 24 hours. No significant differences in the lactate dehydrogenase supernatant level (lactate dehydrogenase cytotoxicity assay; Cayman, Spain) or in the number of apoptotic cells (annexin V-FITC) were observed in comparison with the control group [25].

### Cell stimulations and IL-8 assay

Primary HBECs from non-smoker, smoker, and smokers with COPD were adjusted to 500 × 10^3^ cells per well in 6-well plates and incubated in BEGM culture medium at 37°C with 5% CO_2_. Cells were then treated in the presence or absence of roflumilast N-oxide (0.1 nM-1 μM; Forest Research Institute, Jersey City, USA), dexamethasone (0.1 nM-1 μM; Sigma Aldrich, Madrid, Spain), or with a combination of fixed concentrations of dexamethasone at suboptimal concentrations (10 nM) and roflumilast N-oxide (1 nM, 10 nM, and 100 nM) for 1 hour. After drug incubations, HBECs were stimulated with the TLR3 agonist poly I:C at 10 μg/mL or with the TLR7/8 agonist CL097 (Invivogene, Toulouse, France) at 4 μg/ml final concentration, as previously outlined [28,29]. Cells were co-incubated with the drugs and the TLR agonists for 24 hours.

In additional experiments, BEAS2B cells were exposed to AIR or CSE 1% for 1 hour and then treated for 1 hour with roflumilast N-oxide (0.1 nM-1 μM), dexamethasone (0.1 nM-1 μM), or with a combination of fixed concentrations of dexamethasone at suboptimal concentration (10 nM) and roflumilast N-oxide (1 nM, 10 nM, and 100 nM). Cells were then stimulated with poly I:C at 10 μg/mL or CL097 at 4 μg/ml for 24 hours.

Roflumilast N-oxide and dexamethasone were dissolved in dimethyl sulfoxide (DMSO) at 10 mM stock concentration. Several dilutions of the stocks were performed with cell culture medium. The final concentrations of DMSO (0.1%) in the cell culture did not affect celular functions. Other chemicals (poly I:C or CL097) were dissolved in médium.

In other experiments Roflumilast N-oxide was added at 0, 1 nM, 10 nM or 1 μM. 0 nM corresponds to vehicle (0.1% DMSO), 2 nM corresponds to the free plasma concentrations (unbound to plasma protein) after repeated, oral, once-daily dosing of roflumilast at the clinical dose of 500 μg/day [30] and at 1 μM roflumilast N-oxide completely and selectively inhibits PDE4 [31]. The half-maximum potency of roflumilast N-oxide to inhibit PDE4 amounts to 2 nM [31].

Supernatants were collected and centrifuged at 120 g for 5 minutes. IL-8 was measured in cell-free supernatant and cellular extracts were utilized to measure mRNA. IL-8 concentration was determined using a commercially available enzyme-linked immunosorbent assay kit for IL-8 (R&D Systems, Nottingham, UK).

### NF-κB (p65) and AP1 nuclear transcription factor measure

BEAS2B cells were treated with or without CSE 1% for 1 hour and exposed to roflumilast N-oxide (10 nM, 1 μM), dexamethasone (10 nM, 1 μM) or the combination of roflumilast N-oxide 10 nM plus dexamethasone 10 nM for 1 hour and stimulated with poly I:C 10 μg/mL at indicated times. Cells were then washed and centrifuged to extract the nuclear protein as previously described [10]. Measurement of nuclear NF-κB (p65) transcription factor expression was performed using a commercially available NF-κB (p65) transcription factor assay kit (Cayman Chemical, MI, USA) and with the ELISA AP1 Chemiluminescence Kit (Signosis, CA, USA).

### Analysis of p38 phosphorylation

BEAS-2B were treated with or without CSE 1% for 1 hour and then exposed to roflumilast N-oxide (10 nM-1 μM), dexamethasone (10 nM-1 μM), or a combination for 1 hour. Cells were then stimulated with poly I:C 10 10 μg/mL for 30 minutes. Total protein was extracted using a lysis buffer consisting of a complete inhibitor cocktail plus 1 mM ethylenediaminetetraacectic acid (Roche Diagnostics Ltd, West Sussex, UK) with 20 mM Tris base, 0.9% NaCl, 0.1% Triton X-100, 1 mM dithiothreitol and 1 μg/ml pepstatin A. The Bio-Rad assay (Bio-Rad Laboratories Ltd., Herts, UK) was utilized for protein quantification to ensure equal protein loading. To quantify the phosphorylation of p38, Surveyor™ IC Immunoassay of p38α phosphorylated at T180/Y182 in cell lysates was employed (R&D Systems Europe, Ltd). Results were expressed as pg of phosphor-p38/μg of total protein.

### Analysis of results

Statistical analysis of results was carried out by parametric or non-parametric analysis as appropriate with *P* < 0.05 considered statistically significant. Non-parametric tests were used to compare results from the lung tissue of non-smoker, smoker, and smokers with COPD. In this case, data were displayed as mean ± standard deviation. For comparisons between two groups, differences were analyzed using the Mann–Whitney *U* test. For comparisons between multiple groups, a nonparametric one-way analysis of variance (Kruskal-Wallis test) was performed and post hoc comparison were performed using the Dunn’s post-hoc test, which generalizes the Bonferroni adjustment procedure. Correlations were analyzed using the Spearman (ρ) correlation analysis.

*In vitro* cell experiments were performed in HBECs from non-smoker, smoker and smokers with COPD and in BEAS2B cells. Results were expressed as mean ± standard error of mean (SEM) of *n* experiments with the normal distribution for each data set confirmed by histogram analyses and Kolmogorov–Smirnov test. Parametric analyses were performed and two-group comparisons were analysed using the two-tailed Student’s paired *t*-test for dependent samples or unpaired *t*-test for independent samples. Multiple comparisons were analysed by one-way or two-way analysis of variance followed by Bonferroni post hoc test.

## Results

### Expression and distribution of TLR3, TLR7 and TLR8 in lung tissue of non-smokers, smokers and smokers with COPD

**Figure 1:**
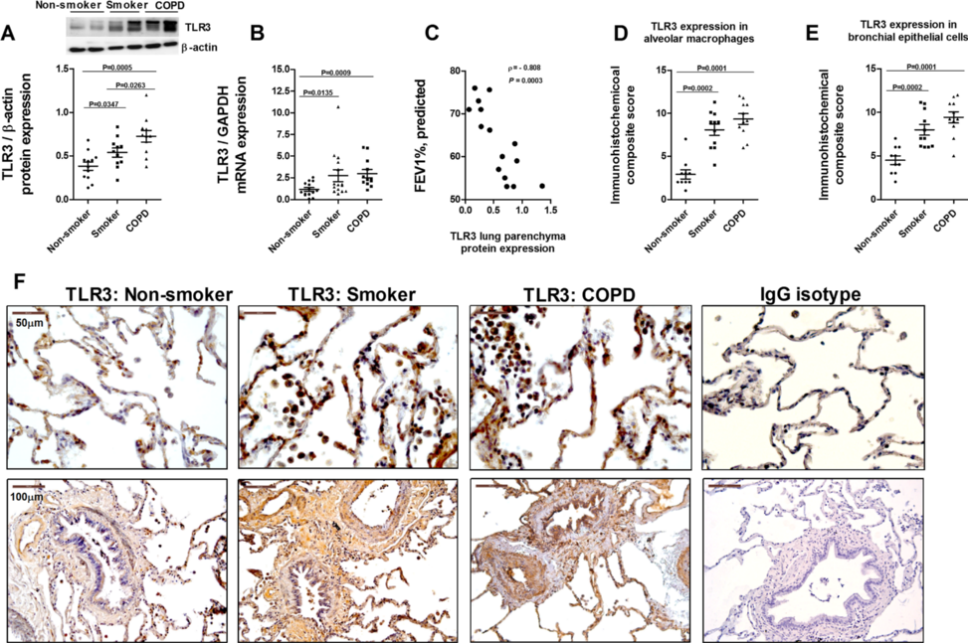
**Expression of TLR3 in lung tissues of non-smokers, smokers, and COPD patients.** Total protein and mRNA was obtained from lung tissue of non-smokers (n = 15), smokers (n = 12), and COPD patients (n = 15). TLR3 protein and mRNA expression was determined by western blot **(A)** and real time PCR **(B)**, respectively, in lung parenchyma. **(A)** Representative images of western blot for TLR3 and corresponding densitometry expressed as ratio of β-actin. **(B)** TLR3 mRNA expression expressed as the ratio to GAPDH. **(C)** Spearman “ρ” correlation of the protein expression of TLR3 in COPD patients and lung function, FEV_1_% predicted. **(D, E, F)** Lung sections were immunostained for TLR3 and quantified by means of immunohistochemical score of TLR3 in alveolar macrophages **(D)** and bronchial epithelial cells **(E)**. **(F)** Representative immunohistochemistry images are shown. The control IgG isotype signal was negative. Data are presented as individual values and mean ± standard deviation. Exact *P* values were obtained using Kruskal-Wallis and Dunn’s post-hoc tests.

**Figure 2:**
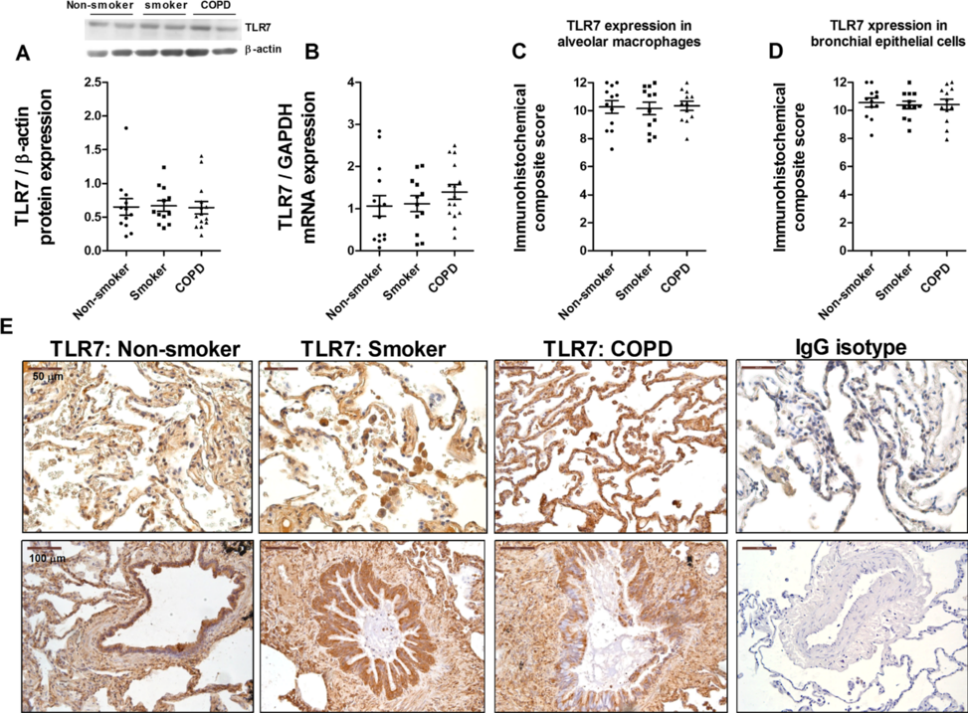
**Expression of TLR7 in lung tissues of non-smokers, smokers, and COPD patients.** Total protein and mRNA were obtained from lung tissues of non-smokers (n = 15), smokers (n = 12), and COPD patients (n = 15). TLR7 protein and mRNA expression were determined by western blot **(A)** and real time PCR **(B)** in lung parenchyma. **(A)** Representative images of western blot for TLR7 and corresponding densitometry expressed as ratio to β-actin. **(B)** TLR7 mRNA expression given as the ratio to GAPDH. **(C, D, E)** Lung sections were immunostained for TLR7 and quantified by means of immunohistochemical score of TLR7 in alveolar macrophages **(C)** and bronchial epithelial cells **(D)**. **(E)** Representative immunohistochemistry images are shown. The control IgG isotype showed negative staining. Data are presented as individual values and mean ± standard deviation. Exact *P* values were obtained using Kruskal-Wallis and Dunn’s post-hoc tests.

**Figure 3:**
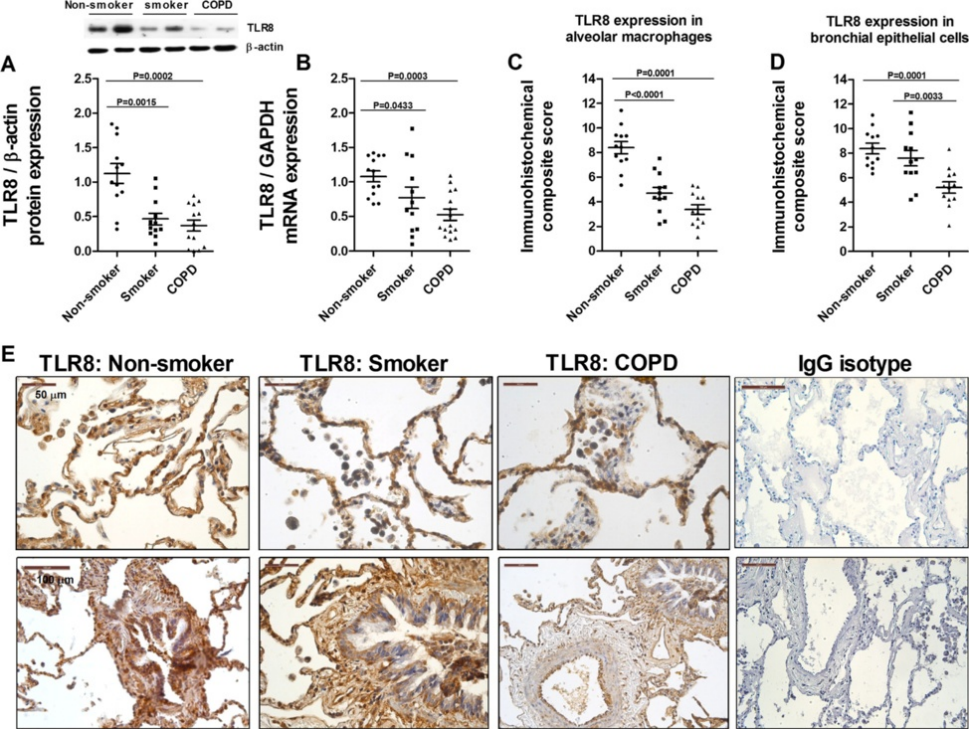
**Expression of TLR8 in lung tissues of non-smokers, smokers, and COPD patients.** Total protein and mRNA were obtained from lung tissues of non-smokers (n = 15), smokers (n = 12), and COPD patients (n = 15). TLR8 protein and mRNA expression were determined by western blot **(A)** and real time PCR **(B)** in lung parenchyma. **(A)** Representative images of western blot for TLR8 and corresponding densitometry expressed as ratio to β-actin. **(B)** TLR8 mRNA expression given as the ratio to GAPDH. **(C, D, E)** Lung sections were immunostained for TLR8 and quantified by means of immunohistochemical score of TLR8 in alveolar macrophages **(C)** and bronchial epithelial cells **(D)**. **(E)** Representative immunohistochemistry images are shown. The control IgG isotype showed negative staining. Data are presented as individual values and mean ± standard deviation. Exact *P* values were obtained using Kruskal-Wallis and Dunn’s post-hoc tests.

TLR3 protein and mRNA expression was upregulated in lung parenchyma of smokers and smokers with COPD and inversely correlated with FEV_1_% in COPD patients (Figure 1A, B, and C). TLR3 immunostaining was significantly elevated in alveolar macrophages and bronchial epithelial cells from smokers and smokers with COPD compared with non-smokers (Figure 1D, E, and F). TLR7 expression was detected in nearly all lung cellular structures, and was similar in lung tissue from non-smokers, smokers, and smokers with COPD (Figure 2). In contrast, TLR8 expression was significantly downregulated in lung parenchyma of smokers and smokers with COPD (Figure 3A and B). While TLR8 showed a marked expression in all lung structures of non-smokers, the immunohistochemical staining of TLR8 showed a weak distribution in alveolar macrophages and bronchial epithelial cells from smokers and smokers with COPD (Figure 3C, D, and E).

### Anti-inflammatory properties of dexamethasone and roflumilast N-oxide on primary human bronchial epithelial cells stimulated with TLR3 agonist

**Figure 4:**
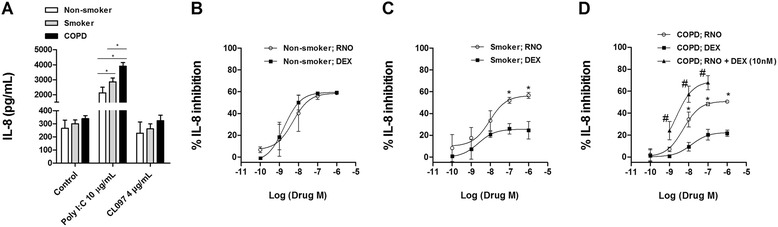
**Roflumilast N-oxide inhibited IL-8 release in human bronchial epithelial cells and demonstrated additive anti-inflammatory properties in corticosteroid resistance conditions.** Human bronchial epithelial cells (HBECs) from non-smokers (n = 4), smokers (n = 4), and COPD patients (n = 5) were isolated from lung tissues. **(A)** HBECs from different patients were stimulated with TLR3 agonist poly I:C or the TLR7/8 dual agonist CL097 for 24 hours and IL-8 levels in the supernatants were measured by ELISA. **(B, C, D)** HBECs from different patients were incubated with roflumilast N-oxide (RNO) or dexamethasone (DEX) at different concentrations for 1 hour followed by the stimulation with poly I:C 10 μg/ml for 24 hours to measure IL-8 release. **(D)** In COPD patients, fixed concentrations of DEX 10 nM were combined with different RNO concentrations. Results are expressed as means ± SEM of *n* = 4–5 (4 non-smoker, 4 smokers, and 5 COPD patients) run in triplicate experiments. Two-way repeated measures analysis of variance (ANOVA) were performed. Post hoc Bonferroni test: *P < 0.05 compared with solvent controls. #*P* < 0.05 compared with monotherapy.

After 24 hours of stimulation with the TLR3 agonist poly I:C 10 μg/mL, the release of IL-8 was increased in HBECs from all patients showing higher amounts in cells from smokers and smokers with COPD. However, the TLR7/8 dual agonist CL097 did not increase IL-8 secretion (Figure 4A).

In HBECs from non-smoker patients, both roflumilast N-oxide and dexamethasone inhibited the poly I:C-induced IL-8 secretion in a concentration-dependent manner with similar maximal percent inhibition (58.8 ± 1.4% and 59.2 ± 1.2% respectively) and potency (−logIC_50_ 8.28 ± 0.43 roflumilast *vs* 8.75 ± 0.21 [M] dexamethasone; Figure 4B and Table 2). While roflumilast N-oxide inhibited IL-8 secretion in cells from smokers and smokers with COPD with similar maximal percent inhibition (56.6 ± 2.8% and 50.5 ± 1.5%, respectively) and potency (−logIC_50_ 8.14 ± 0.21 and 8.28 ± 0.17 [M]), dexamethasone showed impaired maximal percent inhibition in both smokers and smokers with COPD (24.5 ± 7.9% and 21.6 ± 2.8%, respectively) and impaired potency in COPD patients (−logIC_50_ 7.84 ± 1.7 [M]; Figure 4C and D, Table 2).

**Table 2:** **Anti-inflammatory effects of roflumilast N-oxide and dexamethasone in human bronchial epithelial cells**

**A)**		**HBEC; Non-smokers**		**HBEC; Smokers**		**HBEC; COPD**	
**Stimulus**	**Treatment**	**Maximal % inhibition**	**-logIC**_**50**_	**Maximal % inhibition**	**-logIC**_**50**_	**Maximal % inhibition**	**-logIC**_**50**_
**Poly (I:C)** 10 μg/mL	RNO	58.8 ± 1.4	8.28 ± 0.43	56.6 ± 2.8	8.14 ± 0.21	50.5 ± 1.5	8.28 ± 0.17
	DEX	59.2 ± 1.2	8.75 ± 0.21	24.5 ± 7.9*	8.62 ± 0.31	21.6 ± 2.8*	7.84 ± 1.7*^#^
**B)**		**BEAS2B**		**BEAS2B + CSE**			
	**Treatment**	**Maximal % inhibition**	**-logIC**_**50**_	**Maximal % inhibition**		**-logIC**_**50**_	
**Poly (I:C)** 10 μg/mL	RNO	44.5 ± 1.5	8.07 ± 0.15	44.9 ± 4.7		8.08 ± 0.23	
	DEX	42.6 ± 2.9	8.06 ± 0.10	27.9 ± 6.7_┸_		7.77 ± 0.28_┸_	

Inhibition of IL-8 release from (A) human primary bronchial epithelial cells (HBECs) from non-smokers (n = 4), smokers (n = 4) and chronic obstructive pulmonary disease patients (COPD; n = 5) or (B) BEAS2B cells exposed with or without cigarette smoke extract (CSE 1%). (A) Cells were incubated with Roflumilast N-oxide (RNO; 0.1 nM- 1 μM) or Dexamethasone (DEX; 0.1 nM- 1 μM) for 1 hour and stimulated with Poly (I:C) 10 μg/ml (TLR3 agonist) for 24 hours. (B) Cells were incubated with or without CSE 1% for 1 hour, followed by incubation with RNO or DEX for 1 hour and stimulated with Poly (I:C) 10 μg/ml for 24 hours. Values are mean ± SEM of 3–5 independent experiments run in triplicate. IC_50_ values for half-maximum inhibition were calculated by nonlinear regression analysis.*P < 0.05 *vs* RNO group; ^#^ *P* < 0.05 *vs* non-smoker and smoker group; _┸_ *P* < 0.05 *vs* non-CSE treated group.

In HBECs from COPD patients, the combination of suboptimal concentrations of dexamethasone (10 nM) with different concentrations of roflumilast N-oxide (1 nM to 100 nM) additively increased the inhibitory effect of dexamethasone on IL-8 release (Figure 4D).

**Figure 5:**
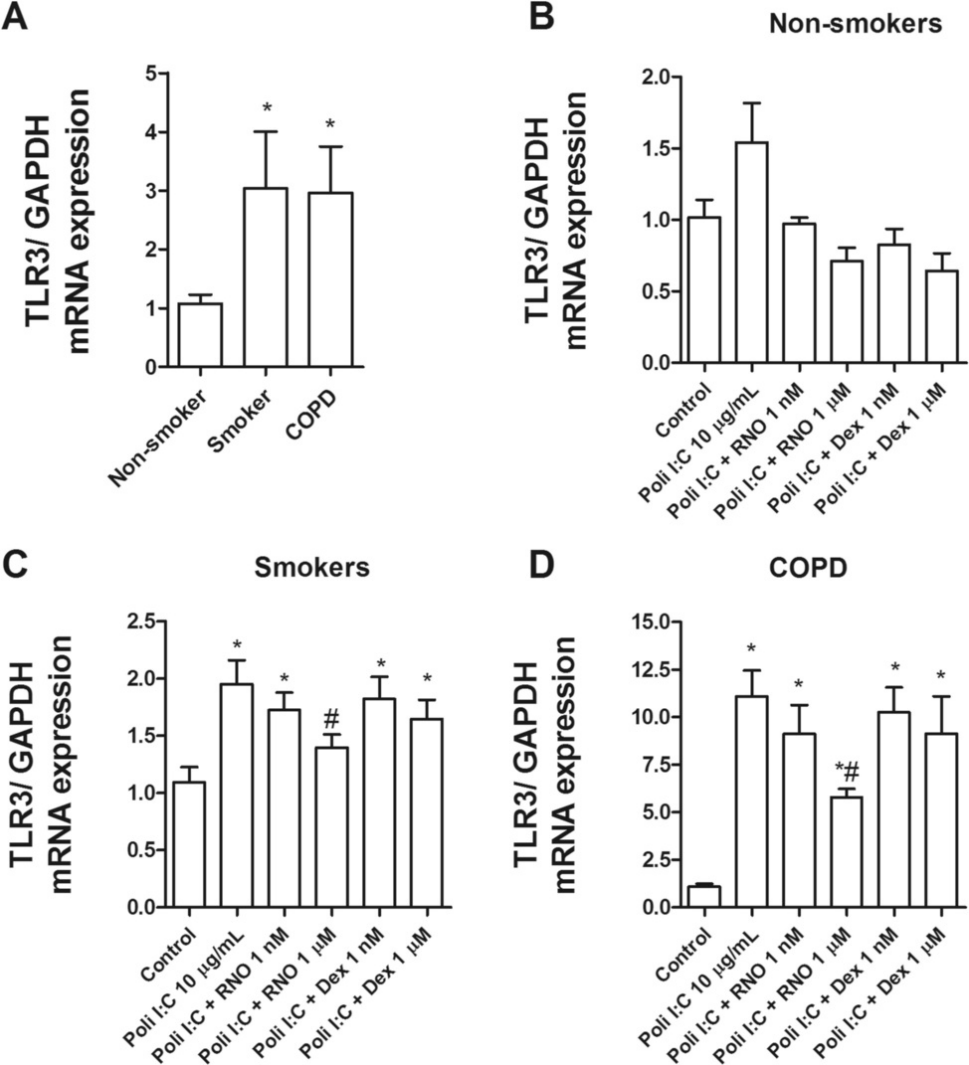
**TLR3 is overexpressed in primary bronchial epithelial cells from current smokers and COPD patients and downregulated by roflumilast N-oxide. (A)** Human bronchial epithelial cells (HBECs) from non-smokers (n = 15), smokers (n = 12), and COPD patients (n = 15) were isolated from lung tissues. **(A)** mRNA expression of TLR3 in HBECs from different patients was determined by real time PCR as the ratio to GAPDH. **(B, C, D)** HBECs from different patients were incubated in the presence or absence of roflumilast N-oxide (RNO) or dexamethasone (DEX) for 1 hour and stimulated with TLR3 agonist poly I:C for 24 hours. Results are expressed as means ± SEM of *n* = 4–5 (4 non-smokers, 4 smokers, and 5 COPD patients) run in triplicate. Two-way repeated measures analysis of variance (ANOVA) were performed. Post hoc Bonferroni test: *P < 0.05 compared with non-smoker group or with solvent controls. #*P* < 0.05 compared with poly I:C stimulus.

**Figure 6:**
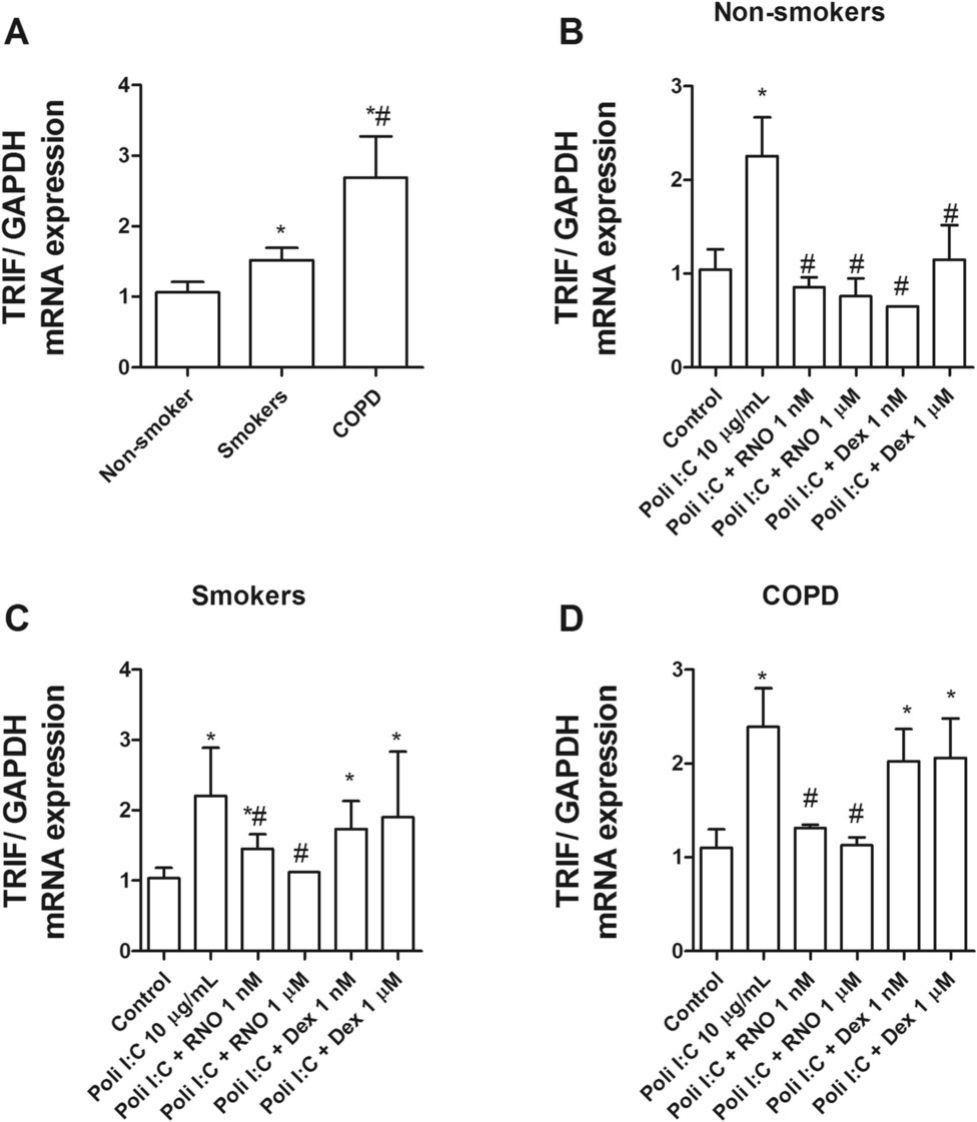
**TRIF is overexpressed in primary bronchial epithelial cells from current smokers and COPD patients and downregulated by roflumilast N-oxide. (A)** Human bronchial epithelial cells (HBECs) from non-smokers (n = 15), smokers (n = 12), and COPD patients (n = 15) were isolated from lung tissue. **(A)** mRNA expression of TRIF in HBECs from different patients was determined by real time PCR as the ratio of GAPDH. **(B, C, D)** HBECs from different patients were incubated in the presence or absence of roflumilast N-oxide (RNO) or dexamethasone (DEX) for 1 hour and stimulated with TLR3 agonist poly I:C for 24 hours to measure TRIF mRNA expression. Results are expressed as means ± SEM of *n* = 3 (3 non-smokers, 3 smokers, and 3 COPD patients) run in triplicate. Two-way repeated measures analysis of variance (ANOVA) were performed. Post hoc Bonferroni test: *P < 0.05 compared with non-smoker group or with solvent controls. #*P* < 0.05 compared with poly I:C stimulus.

Further study of TLR3 revealed a higher expression of TLR3 (Figure 5A) and its adaptor TRIF (Figure 6) in HBECs from smokers and smokers with COPD compared with non-smokers. Interestingly, the TLR3 agonist poly I:C was able to increase significantly the expression of TLR3 (Figure 5B, C, and D) and TRIF (Figure 6B, C, and D) in HBECs from smokers with COPD and to a lesser extent in smokers and non-smoker subjects. Roflumilast N-oxide, reduced the increase of TLR3 and TRIF induced by poly I:C in all conditions. In contrast, dexamethasone only attenuated the increases of TLR3 and TRIF in non-smokers, but not in smokers or smokers with COPD (Figures 5 and 6).

### Cigarette smoke extract reduces corticosteroid responsiveness in BEAS2B bronchial epithelial cells stimulated with TLR3 agonist

**Figure 7:**
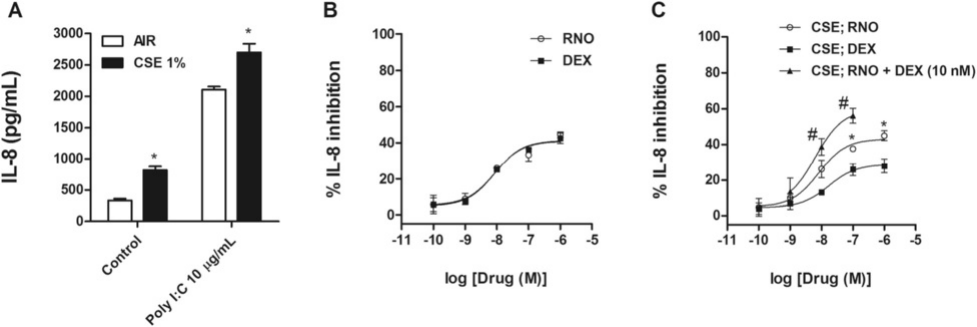
**BEAS2B bronchial epithelial cells exposed to cigarette smoke and stimulated with TLR3 agonist release IL-8 secretion that is inhibited by roflumilast N-oxide but not by dexamethasone.** BEAS2B cells were pretreated **(A, B)** without or **(A, C)** with cigarette smoke extract (CSE) 1% for 1 h followed by the incubation in the presence or absence of different concentrations of roflumilast N-oxide (RNO) or dexamethasone (DEX) for 1 h. After drug incubation cells were stimulated with the TLR3 agonist poly I:C for 24 h and IL-8 release was measured by ELISA. Each graph represents the mean ± SEM of 3–4 independent experiments. One-way repeated measures analysis of variance (ANOVA) were performed. Post hoc Bonferroni test: *P < 0.05 compared with cells exposed to air; #*P* < 0.05 compared with monotherapy.

*In vitro* simulation of smoke exposure was assessed to demonstrate similar behavior of HBECs from smokers and smokers with COPD and to study the underlying mechanistic pathways. In this regard, BEAS2B bronchial epithelial cells were incubated with or without cigarette smoke extract (CSE) 1% for 1 hour, followed by roflumilast N-oxide (0.1 nM-1 μM) or dexamethasone (0.1–1 μM) exposure for 1 hour and the stimulation with poly I:C 10 μg/mL for additional 24 hours to measure IL-8 supernatant levels. CSE exposed cells showed higher IL-8 basal release as well as higher IL-8 amounts following poly I:C stimulation (Figure 7A). In other experiments, cells with or without exposure to CSE did not respond to TLR7/8 agonist CL097 (data not shown).

In absence of CSE pretreatment, both roflumilast N-oxide and dexamethasone concentration-dependently inhibited IL-8 secretion showing nearly identical maximal inhibitory percentage and potency (Figure 7B). In BEAS2B cells pretreated with CSE, roflumilast N-oxide inhibited IL-8 release with comparable maximal inhibitory percentage (44.9 ± 4.7%) and potency (−logIC_50_ 8.08 ± 0.23 [M]; Figure 7C and Table 2) compared with non-exposed cells. In contrast, inhibition of IL-8 by dexamethasone was impaired in the presence of CSE, reducing the maximal inhibitory percentage from 42.6 ± 2.9% to 27.9 ± 6.7% (Figure 7B and C, Table 2).

### Combination of roflumilast N-oxide with dexamethasone shows additive anti-inflammatory effects: mechanistic implications

The combination of a fixed non-effective dexamethasone concentration of 10 nM with different concentrations of roflumilast N-oxide (1 nM–100 nM) in BEAS2B cells pretreated with CSE showed additive effects inhibiting the IL-8 release induced by TLR3 agonist (Figure 7C).

**Figure 8:**
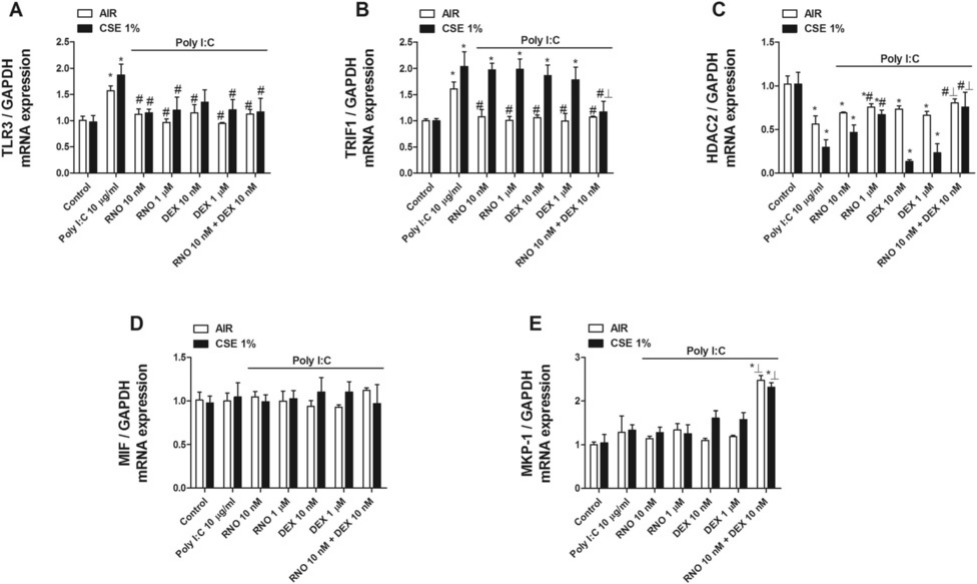
**Roflumilast N-oxide regulation of TLR3 expression and molecular pathway implicated in corticosteroid efficacy.** BEAS2B cells were pretreated with or without cigarette smoke extract (CSE) 1% for 1 hour followed by the incubation in the presence or absence of different concentrations of roflumilast N-oxide (RNO) or dexamethasone (DEX) for 1 hour. After drug incubation cells were stimulated with the TLR3 agonist poly I:C for 24 hours and total mRNA was extracted to quantify the expression of **(A)** TLR3, **(B)** TRIF, **(C)** HDAC2, **(D)** MIF, and **(E)** MKP1 genes by real time PCR. Each graph represents the mean ± SEM of 3–4 independent experiments. One-way repeated measures analysis of variance (ANOVA) were performed. Post hoc Bonferroni test: *P < 0.05 compared with control; #*P* < 0.05 compared with stimulus; _┸_ *P* < 0.05 compared with monotherapy.

Poly I:C in presence or absence of CSE increased the expression of TLR3 and its adaptor TRIF in BEAS2B cells after 24 hours of stimulation (Figure 8A). Roflumilast N-oxide and dexamethasone effectively decreased the poly I:C-induced TLR3 expression in the presence or absence of CSE. However, in the presence of CSE, only the combination of roflumilast N-oxide (10 nM) and dexamethasone (10 nM) inhibited the TRIF overexpression induced by poly I:C (Figure 8B). IL-8 release induced by poly I:C in BEAS2B was insensitive to corticosteroids (Figure 7C). As molecular modulators of corticosteroid efficacy, we found that HDAC2 gene expression was downregulated by poly I:C and further decreased in presence of CSE (Figure 8C). Roflumilast N-oxide at 1 μM partially reversed HDAC2 to control levels, and the association of roflumilast N-oxide 10 nM and dexamethasone 10 nM showed additive effects, increasing HDAC2 expression (Figure 8C). The expression of MIF was not modified under any experimental condition (Figure 8D). The expression of MKP1 was not modified by poly I:C in the presence or absence of CSE and following roflumilast N-oxide or dexamethasone exposure. However, the combination of roflumilast N-oxide 10 nM and dexamethasone 10 nM synergistically increased MKP1 expression (Figure 8E).

**Figure 9:**
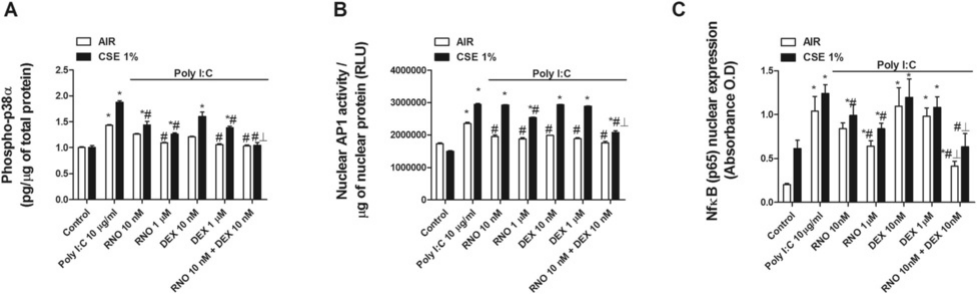
**Roflumilast N-oxide shows additive or synergistic effects with dexamethasone in inhibiting p38, AP1 and NF-**κ**B induced by TLR3 stimulation.** BEAS2B cells were pretreated with or without cigarette smoke extract (CSE) 1% for 1 hour followed by the incubation in the presence or absence of different concentrations of roflumilast N-oxide (RNO) or dexamethasone (DEX) for 1 hour. After drug incubation cells were stimulated with the TLR3 agonist poly I:C for 30 minutes **(A)**, 45 minutes **(B)**, or 1 hour **(C)**, and total protein **(A)** or nuclear protein **(B, C)** was extracted to measure p38 phosphorylation, AP1 nuclear activation, or NF-κB (p65) nuclear expression. Each graph represents the mean ± SEM of 3–4 independent experiments. One-way repeated measures analysis of variance (ANOVA) were performed. Post hoc Bonferroni test: *P < 0.05 compared with control; #*P* < 0.05 compared with stimulus; _┸_P < 0.05 compared with monotherapy.

In other experiments, we observed that TLR3 activation by poly I:C increased the phosphorylation of mitogen-activated protein kinase p38 as well as the nuclear activation of AP-1 and expression of NF-κB (p65) that were enhanced in the presence of CSE (Figure 9A, B and C). Roflumilast N-oxide showed inhibitory effects on phosphorylation of p38 and nuclear activation of AP-1 and NF-κB in the presence or absence of CSE. In contrast, dexamethasone showed a poor inhibitory effect (Figure 8A, B, and C). The combination of roflumilast N-oxide 10 nM and dexamethasone 10 nM synergistically inhibited p38, AP-1, and NF-κB which may explain in part the additive anti-inflammatory effects of roflumilast N-oxide and dexamethasone in HBECs of COPD patients following TLR3 activation.

## Discussion

The present study provides new evidence on the expression and distribution profile of the virus innate immune receptors TLR3, TLR7, and TLR8 in lung tissue of non-smokers, smokers and COPD patients as well as on the anti-inflammatory profile of roflumilast N-oxide and reduced corticosteroid responsiveness following the activation of TLR3 in HBECs from current smokers and COPD patients. Combination of roflimilast N-oxide and dexamethasone showed additive anti-inflammatory properties in HBEC from COPD patients. These results may provide *in vitro* rational for “adding on” a PDE4 inhibitor to the treatment regimen of patients with severe COPD taking inhaled corticosteroids who still suffer frequent exacerbations.

Although TLR3 and, to a lesser extent TLR7/8, have been studied as innate immune viral sensing receptors promoting inflammatory reactions and anti-viral responses in different cell types, their distribution and expression in different lung structures in COPD have been neglected. Among the few available studies, Todt, et al. [19] observed that active smoking, (independent of COPD stage) reduces both the percent of lung macrophages expressing TLR3 and poly I:C-induced CXCL10 production *in vitro*, without altering other endosomal or cytoplasmic receptors such as TLR7/8/9, RIG-I, MDA-5 or PKR, so that positive numbers of TLR3-macrophages directly correlated with lung function. In contrast, Koarai, et al. [17] observed an overexpression of TLR3 in alveolar macrophages of smokers and COPD patients that inversely correlated with lung function. The authors attributed this discrepancy to methodological differences in TLR3 determination, design, and endpoints of the experiments testing polyI:C-stimulated mediator production. Furthermore, a recent work performed by Kinose D, et al. [32] showed an association of the over-expression of TLR3 in sputum cells (mainly neutrophils) with the increase of COPD exacerbations which is in line with the results presented in this work.

To our knowledge, this is the first study which characterizes the main viral pattern recognition receptors TLR3, TLR7, and TLR8 in different lung structures of non-smokers, smokers, and COPD patients. TLR3 was overexpressed in lung parenchyma of current smokers with and without COPD as assessed by western blot, real time PCR, and immunohistochemistry analysis, and inversely correlated with lung function in alveolar macrophages, as previously reported by Koarai, et al. [17]. TLR3 was also overexpressed in different lung structures such as alveolar and bronchial epithelium, as well as in alveolar macrophages of current smokers and COPD patients. Consequently, the stimulation of HBECs from smokers and COPD patients with the TLR3 agonist poly I:C, increased the neutrophilic cytokine IL-8 almost two folds, supporting the hypothesis for which an elevated expression of TLR3 in lung tissue from virally exacerbated COPD patients could mediate inflammatory responses that overwhelm protective anti-inflammatory defenses, and thus plays a role in lung chronic inflammation and remodeling. In fact, a recent study performed in mice deficient in TLR3/7/9 receptor signaling did not exhibit cigarette smoke-induced airspace enlargement, which implicates TLR3 not only in lung inflammation but also in lung remodeling [33]. TLR3 expression is enhanced by dsRNA viral exposure aside from the TLR3 stimulation by its agonist poly I:C and oxidative stress exposure [28,34]. In this work, the TLR3 agonist was able to induce TLR3 and TRIF expression, and cigarette smoke also potentiated TLR3 and TRIF overexpression *in vitro*. However, unlike in non-smokers and smokers, poly I:C increased the TLR3 expression in HBECs from COPD patients by almost 11-fold, that together with the basal over expression of TRIF in HBECs from COPD patient suggest a priming HBECs phenotype which may explain their higher IL-8 release. As a limitation, we included only patients who were free of symptoms of upper respiratory bacterial or virus tract infection. However we cannot discard a chronic subclinical viral bronchial colonization. The presence of non-symptomatic lung viral colonization could stimulate or alter TLR3 expression representing a limitation of this study.

In contrast to TLR3, we did not detect differences of TLR7 expression and distribution in different lung structures, which is in agreement with the observations made in alveolar macrophages of non-smokers, smokers, and COPD patients [19]. However, the expression of TLR8 was decreased in lung parenchyma of smokers and COPD patients as well as in alveolar macrophages and bronchial epithelial cells. In contrast to TLR3, the role of TLR7 and TLR8 in COPD is not well understood. Based in our *in vitro* results, the stimulation of TLR7/8 in HBECs from different patients did not enhance IL-8 secretion. Similar results in airway epithelial cells have been reported previously [28,29] suggesting a less prominent role on airway inflammation for TLR7/8 when compared with TLR3.

The loss of corticosteroid responsiveness is a feature characteristic of severe asthma and COPD [35]. Furthermore, corticosteroids show impaired anti-inflammatory properties following airway viral infection as previously reported [5,6,8,36]. Recent evidence shows that HRV airway epithelial cell infection impaired dexamethasone-dependent inhibition of IL-8 release [6]. Additionally, in an animal asthma model infected with RSV, corticosteroids did not reduce lung inflammation [36]. The loss of corticosteroid responsiveness to lung virus-induced inflammation could be mediated by the activation of TLR3 as mice exposed to poly I:C demonstrated a corticosteroid resistant airway neutrophilia when treated with or without cigarette smoke [8].

In this work we observed that HBECs from current smokers and COPD patients stimulated with TLR3 agonist were insensitive to the anti-inflammatory effects of dexamethasone. These results were also reproduced *in vitro* in BEAS2B cells pretreated with cigarette smoke and stimulated with poly I:C.

Oxidative stress induced by cigarette smoke or chronic inflammation is known to induce corticosteroid resistance. For example, we and others have shown that cigarette smoke may induce inflammation resistant to corticosteroids in different cell types relevant to COPD such as neutrophils [10], HBECs [37], or alveolar macrophages [35]. The increase of oxidative stress generated by cigarette smoke alters mechanistic pathways related with corticosteroid activity. It has been shown that cigarette smoke can phosphorylate mitogen-activated protein kinases such as p38, JUN or ERK1/2, thus promoting glucocorticoid receptor (GR) hyperphosphorylation and consequently the inhibition of GR nuclear translocation [38]. Similar results have been observed in corticosteroid resistant HRV infected airway epithelial cells [6]. Other cellular mechanisms that promote corticosteroid resistance include decreased corticosteroid-induced mitogen-activated protein kinase phosphatase 1 (MKP1) gene expression, NF-κB over activation, or HDAC2 downregulation [38]. We have previously demonstrated that neutrophils from COPD patients are insensitive to corticosteroids and deficient in MKP1 and HDAC2 expression and activity. Furthermore, dexamethasone did not inhibit cigarette smoke-induced NF-κB in neutrophils from COPD patients [10]. In a similar way, airway epithelial cells infected by HRV impaired dexamethasone-induced MKP1 gene expression, diminished binding of GR to glucocorticoid response element (GRE), and impaired GR nuclear translocation as well as NF-κB over activation and GRα hyperphosphorylation [6].

In this work, we observed that HBECs from smokers and smokers with COPD stimulated with poly I:C increased the TLR3 and TRIF expression which was not inhibited by dexamethasone. Additionally, the bronchial epithelial cells exposed to cigarette smoke and stimulated with TLR3 agonist induced HDAC2 downregulation and an increase in p38 phosphorylation and AP1 and NF-κB nuclear expression that were poorly inhibited by dexamethasone.

PDE4 inhibitors have shown potent anti-inflammatory properties in several cell types implicated in COPD pathogenesis (with the exception of alveolar macrophages which show a low PDE4 expression [39,40]), and can decrease the cellular oxidative stress generated by cigarette smoke and RSV as we previously outlined [3,9,23]. Additionally, the combination of the PDE4 roflumilast with corticosteroids has shown additive or synergistic properties on the activation of anti-inflammatory genes and the inhibition of proinflammatory cytokines [10,41,42]. In fact, roflumilast increased the GRE signal induced by corticosteroids in bronchial epithelial cells and increased expression of the anti-inflammatory genes induced by GRE [41]. In neutrophils from COPD patients, roflumilast showed potent anti-inflammatory properties and the combination of roflumilast with suboptimal dexamethasone concentrations also increased the anti-inflammatory properties of dexamethasone. The exploration of the underlying mechanisms revealed that roflumilast enhanced the ability of dexamethasone to increase HDAC2 activity and MKP1 expression as well as to inhibit ERK1/2 phosphorylation and NF-κB nuclear expression. In a recent study, we explored the anti-inflammatory role of roflumilast on the dsRNA RSV infection and inflammation [9]. Roflumilast inhibited RSV infection, prevented the loss of cilia and the secretion of proinflamatory IL-13, IL-6, IL-8, and TNFα, and decreased formation of ROS. This suggests an anti-inflammatory role of roflumilast in similar conditions to those observed in corticosteroid resistance. In the present work, in contrast to dexamethasone, we observed an inhibition of the TLR3 and TRIF expression by roflumilast N-oxide in HBECs from smokers and smokers with COPD. Furthermore, roflumilast N-oxide in combination with dexamethasone showed additive inhibition of IL-8 release in HBECs from smokers and smokers with COPD. This association synergistically increased MKP1 and HDAC2 expression in bronchial epithelial cells pretreated with cigarette smoke and stimulated with poly I:C. Similar findings were also observed in neutrophils from COPD patients that were stimulated with cigarette smoke [10]. Furthermore, roflumilast N-oxide in combination with dexamethasone also showed additive inhibitory effects on p38 phosphorylation and AP1 and NF-κB nuclear expression.

As previously reported, corticosteroid sensitivity was restored by inhibiting NF-κB in infected HRV airway epithelial cells [6] which supports our findings on roflumilast N-oxide in cells stimulated with TLR3 agonist.

TLR3 agonist and CSE combination induced resistance to dexamethasone, however we do not tested whether the IL-8 increase in BEAS2B due to CSE alone is reversible by roflumilast, dexamethasone or their combination. In this regard, it has been shown previously that CSE reduced the increase of GRE nuclear binding induced by dexamethasone in bronchial epithelial cells, thus impairing its anti-inflammatory properties [43]. In contrast, we previously have observed anti-inflammatory properties of roflumilast in human bronchial epithelial cells stimulated with CSE (data not shown). Whether the combination of roflumilast and dexamethasone reverse corticosteroid resistance induced by CSE alone, remains to be explored, although the additive/synergic effects of roflumilast and dexamethasone on GRE activation in absence of oxidative stress has been observed [41] suggesting similar results under oxidative stress conditions. Besides the utility of using BEAS2B cell line as a mechanistic model of corticosteroid resistance, we have to highlight that BEAS2B cells could respond differently to stimuli compared to primary cells, which represents an important limitation of this study.

Clinically there may be a scientific rationale for using roflumilast in combination with an inhaled corticosteroid. In a recent post hoc analysis of two phase III studies, roflumilast reduced exacerbation frequency in a subgroup of patients with severe COPD and chronic bronchitis who were taking an inhaled corticosteroid concomitantly [21].

Moreover, roflumilast is recommended for patients with severe disease who are already taking a LABA/inhaled corticosteroid combination. Although not tested in this work, the triple combination of LABA/inhaled corticosteroid/roflumilast has shown potent synergistic anti-inflammatory properties [41]. Frequent exacerbations of COPD are associated with a high level of inflammation [44] that may be less sensitive to corticosteroids, thus the combination of these anti-inflammatory therapies may be more effective in reducing COPD exacerbations. Anyway, clinical translation of our findings are far from resolved.

## Conclusions

The present work shows that TLR3 expression is up-regulated in lung tissue from smokers and smokers with COPD correlating inversely with lung function, while TLR8 is down-regulated and TLR7 remains unaffected. TLR3 over-expression triggered IL-8 release in human bronchial epithelial cells from smokers and smokers with COPD patients as well as in cells exposed to CSE, that was insensitive to corticosteroids but not to roflumilast N-oxide suggesting a prominent role of cigarette smoke on corticosteroid insensitivity. Combination of roflumilast N-oxide with dexamethasone showed additive anti-inflammatory effects that provide *in vitro* evidence for a possible clinical utility to add roflumilast on top of inhaled corticosteroid in severe COPD.

## Abbreviations

AP1: Activator protein 1
COPD: Chronic obstructive pulmonary disease
CSE: Cigarette smoke extract
GOLD: Global initiative for chronic obstructive lung disease
HBEC: Human bronchial epithelial cells
HDAC2: Histone deacetylase 2
HRV: Human rhinovirus
IL-8: Interleukin 8
LABA: Long-acting beta agonist
LAMA: Long-acting muscarinic antagonist
MIF: Macrophage migration inhibitory factor
MKP1: Mitogen-activated protein kinase phosphatase–1
NF-κB: Nuclear factor kappa B
PDE4: Phosphodiesterase–4
ROS: Reactive oxygen species
RSV: Respiratory syncytial virus
TLR: Toll like receptor
